# Investigation of the Effects of Cardiovascular Therapeutic Ultrasound Applied in Female and Male Rats’ Hearts of Different Ages

**DOI:** 10.1109/TUFFC.2021.3113867

**Published:** 2021-12-31

**Authors:** Olivia C. Coiado, Rahul S. Yerrabelli, Anton P. Christensen, Marcin Wozniak, William D. O’Brien

**Affiliations:** Carle Illinois College of Medicine, Champaign, IL 61801 USA; Bioacoustics Research Laboratory, Department of Electrical and Computer Engineering, University of Illinois at Urbana–Champaign (UIUC), Urbana, IL 61801 USA.; Carle Illinois College of Medicine, Champaign, IL 61801 USA; Bioacoustics Research Laboratory, Department of Electrical and Computer Engineering, University of Illinois at Urbana–Champaign (UIUC), Urbana, IL 61801 USA.; Carle Illinois College of Medicine, Champaign, IL 61801 USA; Bioacoustics Research Laboratory, Department of Electrical and Computer Engineering, University of Illinois at Urbana–Champaign (UIUC), Urbana, IL 61801 USA.; Beckman Institute for Advanced Science and Technology, University of Illinois at Urbana–Champaign (UIUC), Urbana, IL 61801 USA; Department of Medical Laboratory Diagnostics-Biobank, Medical University of Gdańsk, 80-210 Gdańsk, Poland.; Bioacoustics Research Laboratory, Department of Electrical and Computer Engineering, University of Illinois at Urbana–Champaign (UIUC), Urbana, IL 61801 USA.

**Keywords:** Biological effects, high-power ultrasound (US), therapeutics

## Abstract

This study investigates the role of age and sex on the cardiovascular effects of 3.5-MHz pulsed ultrasound (US) in a rat model. Ultrasonic bursts of 2.0-MPa peak rarefactional pressure amplitude (equivalent to an *in vitro* spatial-peak temporal-peak intensity of ~270 W/cm^2^ and a mechanical index of 1.1) were delivered in five consecutive 10-s intervals, one interval for each pulse repetition frequency (PRF) (6, 5, 4, 5, and 6 Hz; always the same order) for a total exposure duration of 50 consecutive seconds. Sixty F344 rats were split into 12 groups in a 3 × 2 × 2 factorial design (three ages, male versus female, and US application versus control). This study is the first study on US-induced cardiac effects that contains data across three age groups of rats (premenopause, fertile, and postmenopause) to mimic the fertile and nonfertile human window. US was applied transthoracically, while heart rate, stroke volume, ejection fraction, temperature, and other physiologic parameters were recorded at baseline and after exposure. Significant decreases in cardiac output compared to respective control groups were observed in multiple experimental groups, spanning both females and males. A negative chronotropic effect was observed in young male (~7%) and female (~16%) rats, in five-month-old male (~9%) and female (~15%) rats, and in old rats where the effect was not statistically significant. Younger groups and, to a lesser extent, lower weight groups generally had more significant effects. The pathophysiology of US-induced cardiovascular effects appears to be multifactorial and not strictly related to hormones, menopause, weight, sex, or age, individually.

## I. Introduction

The goal of this article is to evaluate the feasibility of using pulsed ultrasound (US) as a therapeutic and noninvasive treatment for cardiovascular disease (CVD). In addition, this study aims to explain the role of this new US therapy in hormonal differences, evaluating premenopausal and postmenopausal female rats and male rats of different ages. The motivation of this study and long-term goal is to potentially use this alternative therapy as a substitute for pacemakers and eliminate complications caused by the electrodes, broken leads, infections, and so on.

CVD is the leading cause of death worldwide, being responsible for an estimated 31% of deaths globally [1]. CVD manifests itself as an increase in blood pressure, which can injure the heart and affect its function. The increased blood pressure can be due to varying causes, such as atherosclerosis of arteries, vasculitis, underlying biochemical changes, or deficiencies in the heart itself [2].

Blood pressure is higher in men than in women, and women experience a delay in the onset of CVD in comparison to men and believed to be due to an interplay between levels of both androgens and estrogens [3]-[6]. Androgens are hypothesized to increase blood pressure in different ways from contributing to vasoconstriction [7], upregulating the renin–angiotensin system (RAS), inducing oxidative stress, retaining more Na+, and decreasing nitric oxide levels [3]. Estrogens on the other hand are hypothesized to have a protective effect against atherosclerosis and CVD [8]. Estrogens, such as 17*β*-estradiol, have been shown to stimulate nitric oxide synthase mediating smooth muscle relaxation in blood vessels [8]. The 17*β*-estradiol-mediated vasodilation has also been shown to mediate decreases in core temperature [9].

Clinical studies have shown that women who undergo hysterectomies have a higher prevalence and incidence of both CVD and increased blood pressure that equals or surpasses age-matched men [3]. This is indicative of the protective role that ovarian hormones play against CVD [10].

There are also gender differences in complications of CVD, such as arrhythmias [11], [12]. Cardiac arrhythmias are abnormal contractions in the heart due to dysfunction in the heart’s electrical activity [13]. Clinical studies on arrhythmias have shown that women experience a greater prevalence of sick sinus syndrome and atrial fibrillation with bradycardia, while men exhibited higher rates of atrioventricular (AV) node block [14]. Gender differences persist when comparing outcomes of arrhythmias treated with cardiac pacemakers [14]. While there were fewer favorable outcomes for women (more implantation complications, especially pocket hematoma and pneumothorax), women had a significantly longer survival time than men after pacemaker implantation. This is true despite women having a markedly higher age at implantation.

In addition, male sex was a predictor of increased mortality in a long-term follow-up study [14]. Pacemakers were developed over 50 years ago and have been successful in treating many types of arrhythmias. Worldwide annually around 1 million people are implanted with pacemakers [15]. In short-term follow-up, there are 12% complications, and another 3.5% will be noted in long-term follow-up [16]. Complications that may occur during surgery include allergic reactions, infections, vessel damage, and heart tissue punctures [17]. The weakest link in the pacemaker system, most often leading to complications, is the lead. In contrast to traditional pacemakers, ultrasonic pacing could serve as a completely extracorporeal device, potentially a wearable pacemaker [18], [19]. Furthermore, serious complications, including vascular rupture, infection, and need for extraction, could be avoided or at least dramatically reduced. Cardiac pacing using pulsed US is clinically justified and can be utilized as an alternative and truly noninvasive heart rate (HR) modulator.

In 1929, Harvey [20] indicated the opportunity of US in cardiac pacing. Harvey’s experiments revealed that after using 340-kHz US, reptile and amphibian hearts began to vibrate. Decades after this discovery, studies on frogs showed changes in HR and aortic pressure as a result of US pulses, suggesting the feasibility of US in cardiac stimulation [21], [22]. In 2017, Coiado and O’Brien, Jr., [25] extensively evaluated in rat hearts the negative chronotropic effect (i.e., the decrease of HR via transthoracic US) as well as identifying marked arrhythmias. Over the past 88 years, this study is the only attempt to translate this technology to humans.

Since elevated HR and consequentially blood pressure increases risk for CVD, negative chronotropic pacing could theoretically decrease risk of CVD. However, in the absence of heart failure or coronary artery disease (CAD), treating hypertensive patients with heart-rate-lowering medications has not been shown to reduce adverse events when compared to control [23]. Negative chronotropic pacing also shows promise as an adjunct or alternative therapy for cardiac arrhythmias [24]. Due to the particular effectiveness of pulsed US in female rats and the increased rate of atrial fibrillation in women, pulsed US could play a vital role in the future treatment of cardiac arrhythmias [25].

## II. Materials and Methods

### A. Animals, Preparation and Measurements Systems

The experimental protocol for this study was approved by the University of Illinois Institutional Animal Care and Use Committee (Protocol #10104). A total of 60 F344 rats (Harlan, Indianapolis, IN) were evenly divided into 12 groups of five rats in a 3 × 2 × 2 factorial design: 1) age (three-month versus five-month versus 24-month); 2) male versus female; and 3) US-on (exposure) versus US-off (control). The rat weights were 150–200 g (three-month female), 170–200 g (five-month female), 250–300 g (24-month female), 300–350 g (three-month male), 350–410 g (five-month male), and 400–500 g (24-month male). The three- and five-month rats are at fertile (but post-pubescent) age and the 24-month-old female rats are after reproductive senescence (“menopause”) [26]. Preliminary data on the 3- and 24-month rats were included in a prior study [25].

The anesthesia, preparation, and measurement techniques paralleled our previous studies [24], [25], [27]. All rats (including control) were anesthetized with 5% isoflurane for the initial induction of anesthesia and then received 1.5%–2% isoflurane for maintenance, with monitoring anesthesia levels via pedal reflex. Notably, while isoflurane does not impact cardiac contractility, it can act as a respiratory depressant and thereby affect cardiac parameters [28], [29]. However, like the experimental rats, the control rats were exposed to the anesthesia and all other steps of the protocol, except for US application, so any anesthesia-induced effects would appear in both groups. In addition, the time to experiment was minimized (~2 h for each animal, time from applying the anesthesia to completion of experiments).

**Fig. 1.**
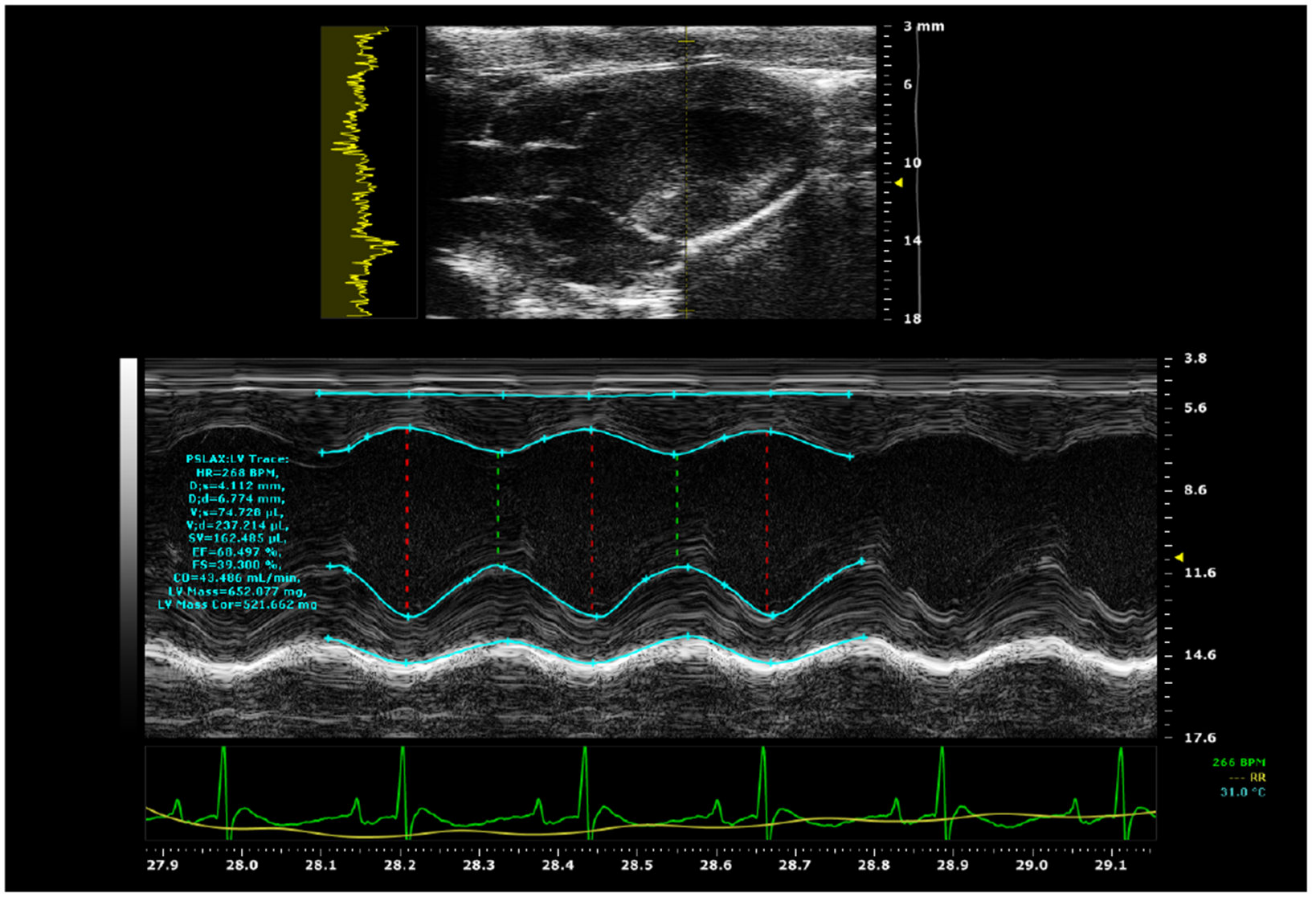
B-mode (top) and M-mode (bottom) images of a rat heart (three-month-old female rats) 15 min after US exposure.

To maximize transthoracic acoustic transmission, the thoracic area of the rats was shaved and depilated and gel was used for acoustic coupling. Animals were placed on a temperature-controlled platform in a dorsal recumbency position for US cardiac exposure. The rats’ limbs were secured to the four-lead electrocardiogram (EKG) pads on the animal platform, a capability connected to the small animal Vevo 2100 high-frequency (13–24 MHz) US imaging system (VisualSonics, Toronto, ON, Canada) so that the physiological data of the rats could be monitored in real time and could be recorded. The Vevo 2100 was used to monitor the heart via B-mode and M-mode (Fig. 1) displays by a registered diagnostic medical sonographer (RDMS).

Cardiac parameters [i.e., ejection fraction (EF) and stroke volume (SV)] were calculated by the ventricular trace tool of the Vevo 2100 workstation, which can trace the position of the inner and outer ventricular walls on a long-axis M-mode tracing of the left ventricle. The left ventricular internal diameter during systole and diastole was used to calculate the end-systolic volume (ESV) and the end-diastolic volume (EDV), respectively, by the Teichholz method. Subsequently, ESV and EDV were used to calculate the SV and EF [24].

For the continuous measure of arterial pressure, the left femoral artery was cannulated and connected to a control unit (Samba 201, Samba Sensors, Gothenburg, Sweden) and a pressure transducer, low-pressure range (−50 to +350 mbar) (Samba Preclin 420lP, Samba Sensors, Gothenburg, Sweden). Arterial pressure is useful for the detection of potential changes that might cause reflex responses related to the heart. In addition, the rectal temperatures (RTs) of all rats were continuously monitored. Temperature also has utility as a surrogate for hormonal change [9], [30]. Intrathoracic temperature measurement was avoided due to invasiveness. Approximately 18 min after US exposure ceased, the rats were euthanized (5 min of CO_2_ inhalation) for the histological evaluation of the lung and heart by a board-certified pathologist.

### B. Ultrasound Application

An ultrasonic transducer with an unfocused 19-mm-diameter aperture and a center of frequency of 3.5 MHz was used in this experiment (Valpey Fisher, Valpey Corporation, Hopkinton, MA, USA). From previous studies [24], [25], we concluded that the ultrasonic frequency threshold for a bioeffect varies from 67 kHz up to 3.5 MHz. Calibration of the transducer was performed in a tank of distilled, degassed 22 °C water. The calibrated hydrophone was a polyvinylidene fluoride (PVDF) membrane hydrophone (Y-34–3598 EW295, GEC Marconi, Chelmsford, U.K.) with an active element that was 0.5 mm in diameter. The transducer was fixed in place, while the hydrophone was moved by a micropositioning system (2-*μ*m translational accuracy) in a direction perpendicular to the beam axis at a distance of 1 cm from the transducer surface (in the near field) [31].

The ultrasonic transducer was driven by a radio frequency (RF) power amplifier (A150, Electronic Navigation Industries, Rochester, NY, USA; 0.3–35 MHz; 55 dB) and a function generator (33250A, Agilent Technologies Inc., Santa Clara, CA, USA).

After several studies [24], [25], [27], we concluded that the ideal US transducer center frequency can range from 67 kHz up to 3.5 MHz (maximum), pulse repetition frequency (PRF) can vary slightly below or above 5 Hz, the peak rarefaction pressure amplitude (PRPA) maximum can be 2.5 MPa, and the duty cycle is ~0.5%–1%. The PRF sequence can start slightly below or above the HR of the rat (for rats, the HR is 300–350 beats/min or ~5–6 Hz) and decreasing/increasing by 1-Hz steps; this sequence was designed to achieve cardiac pacing while minimizing tissue heating (50-s total ultrasonic exposure duration).

**Fig. 2.**
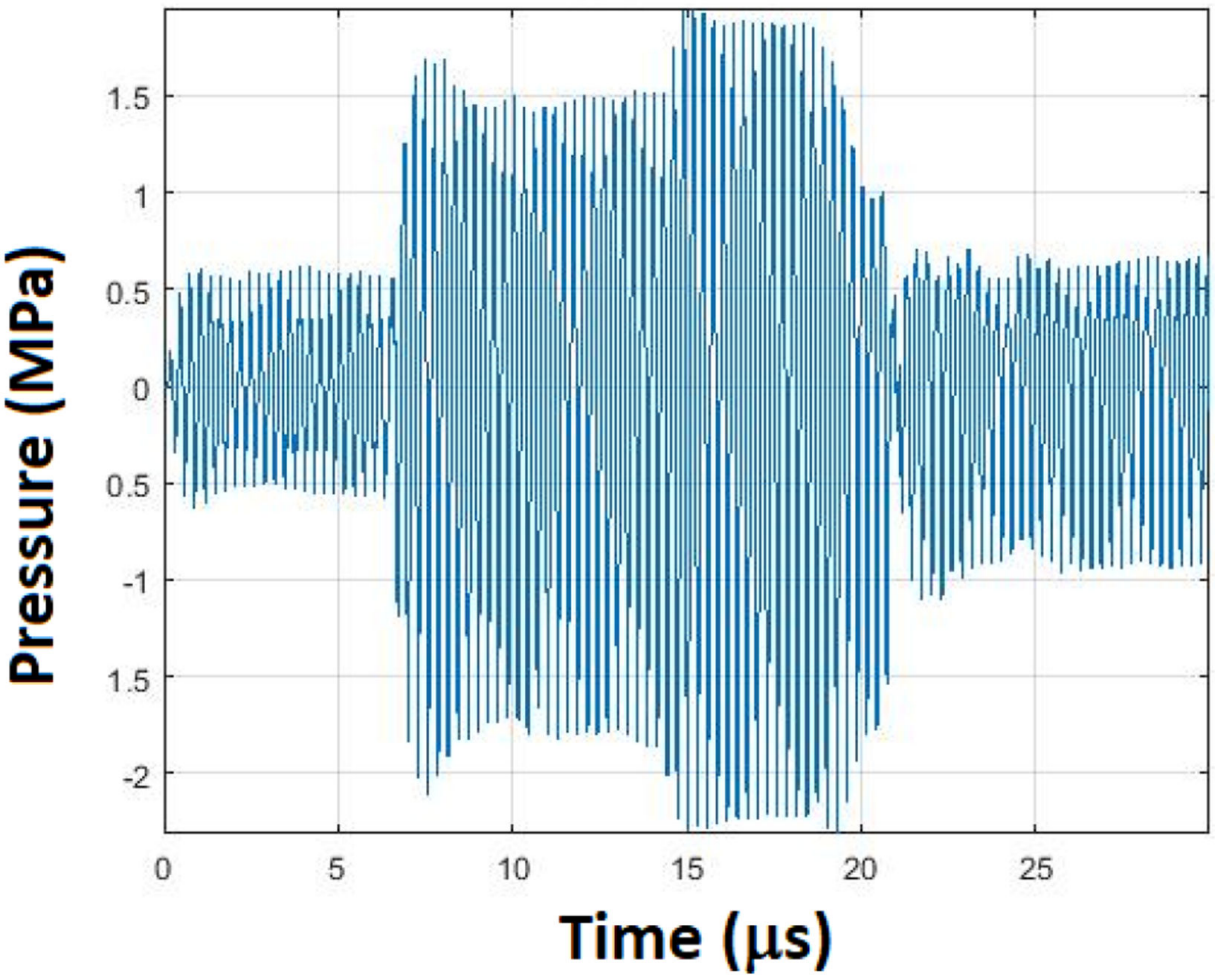
Transducer pressure response at ~2 MPa.

Ultrasonic bursts of 2.0-MPa peak rarefactional pressure amplitude (Fig. 2) (equivalent to an *in vitro* spatial-peak temporal-peak intensity of ~270 W/cm^2^ and a mechanical index of 1.1 [25]) were delivered in five consecutive 10-s intervals, one interval for each PRF (6, 5, 4, 5, and 6 Hz; always the same order) for a total exposure duration of 50 consecutive seconds. Thus, the PRF sequence started slightly above the natural rate HR (approximately 300–350 beats/min or 5–6 Hz), decreasing and increasing in increments of 1 Hz. The duty factor was approximately 1.0%, which indicates that for a PRF sequence of 6, 5, 4, 5, and 6 Hz (167-, 200-, 250-, 200-, and 167-ms pulse repetition periods, respectively), the pulse duration (PD) was 2 ms. From previously published model estimates of this protocol, the steady-state *in situ* temperature increase was estimated to be 0.89 °C [25]. In the control rats, the exact same protocol was performed for animal preparation, US application, and parameter recording, except that the US transducer was turned off while placing the transducer on the rats’ thoracic area.

### C. Statistical Analysis

The data points collected 3 and 15 min after (collectively called the post-US exposure time points, even for the control groups) each rat were divided by the corresponding baseline (pre-US exposure, at time = 0) value to yield a normalized *d* change for each rat. Temperature values were converted to kelvin to have chemical meaning when dividing temperatures. In each of the six demographic groups (two sexes and three ages), an unpaired Student’s *t*-test (*N* = 5 control and experiment, each) was used to determine significance between the experimental and control subgroups. This was done for both of the post-US exposure time points (3- and 15-min post-US relative to baseline) and each of the major parameters including HR, cardiac output (CO), stroke (systolic) volume, EF, EDV, ESV, respiratory rate, and arterial pressure (total of 10 × 2 = 20 comparisons for each of the six demographics). Two-way analysis of variance (ANOVA) for repeated measures was performed among sex, age, and US for each of the parameters and at 3- and 15-min post-US exposure to determine which effects (sex, age, and/or US) were significant across the entire dataset and if there were an interaction.

Statistical analyses and figures were generated in R (version 3.6.1, “Action of the Toes,” The R Foundation for Statistical Computing, Vienna, Austria). Results are expressed as mean and standard error of the mean (SEM). The significance level was set at 0.05. The notation of variable followed by time (i.e., HR3, SV0, and EF15) is used to represent the values at a time point (i.e., HR at 3-min post-US, SV at baseline, and EF at 15-min post-US).

## III. Results

**Fig. 3.**
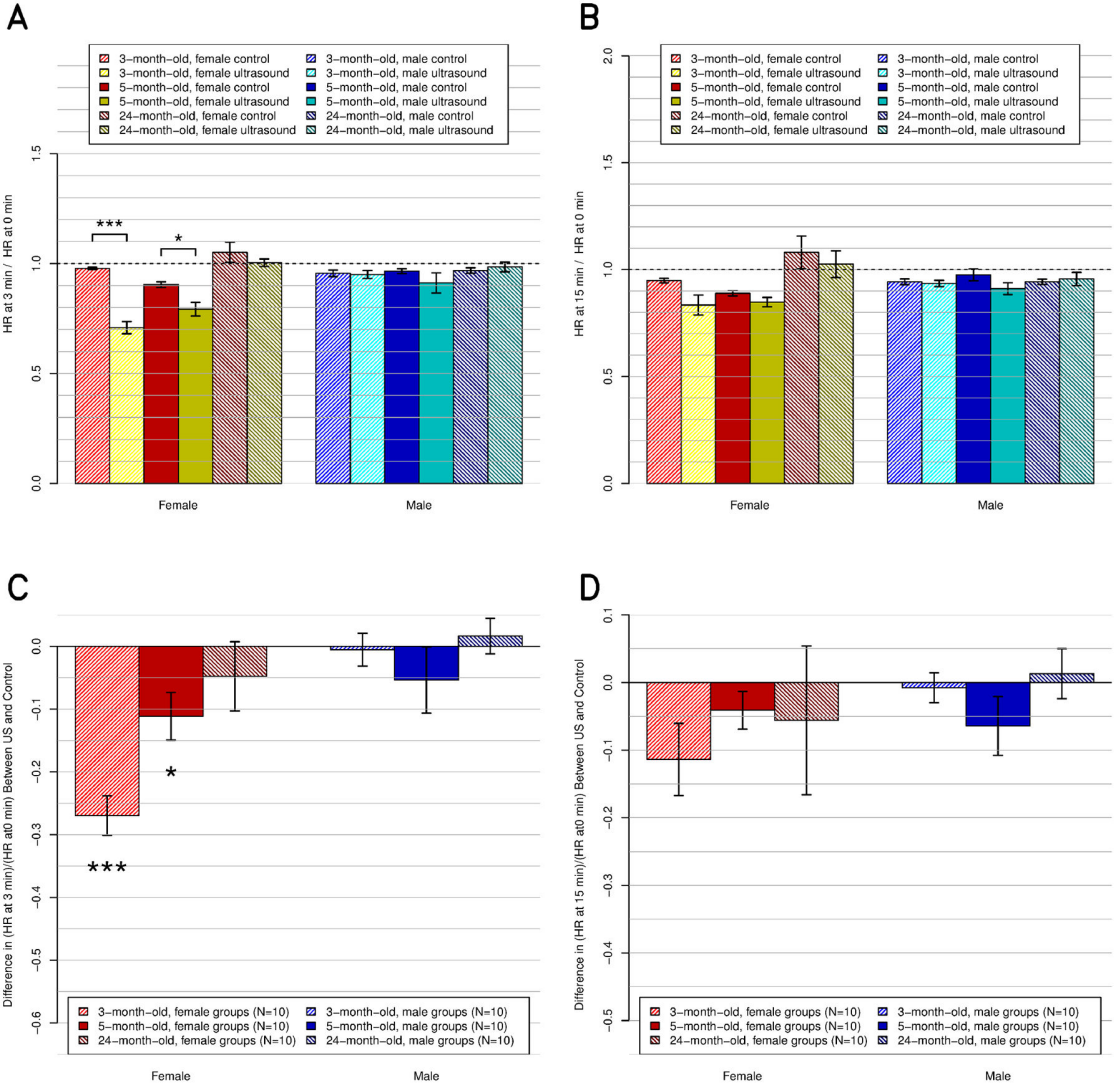
HR, relative to preultrasound baseline, at the postultrasound times of (a) and (c) 3 and (b) and (d) 15 min. (a) and (b) All 12 subgroups individually (*N* = 5 ea.), including the six control and six experimental subgroups. (c) and (d) Same data but present the difference between the experimental subgroups and their respective control subgroups.

**Fig. 4.**
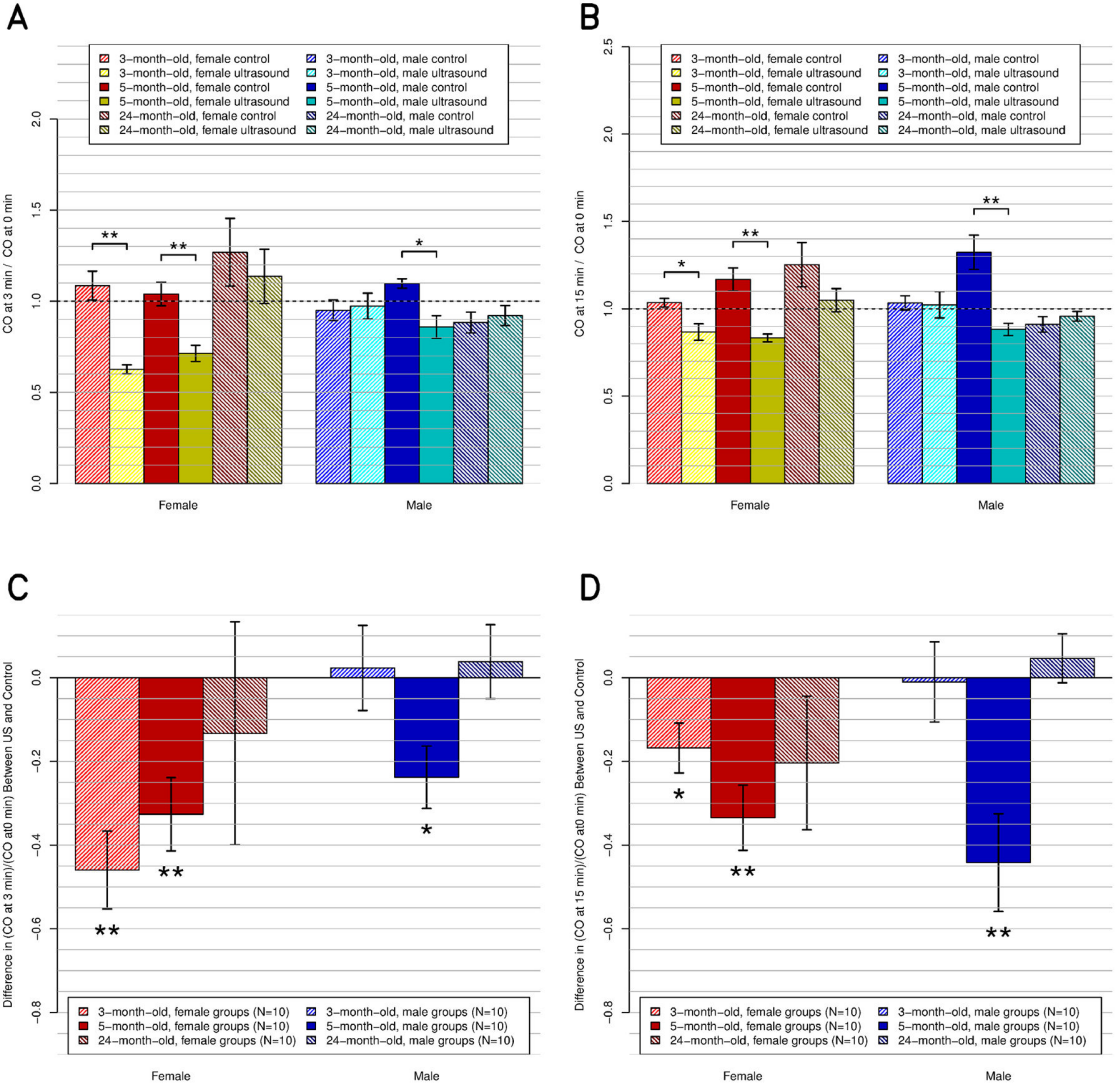
CO, relative to preultrasound baseline, at the postultrasound times of (a) and (c) 3 and (b) and (d) 15 min. (a) and (b) All 12 subgroups individually (*N* = 5 ea.), including the six control and six experimental subgroups. (c) and (d) Same data but present the difference between the experimental subgroups and their respective control subgroups.

**Fig. 5.**
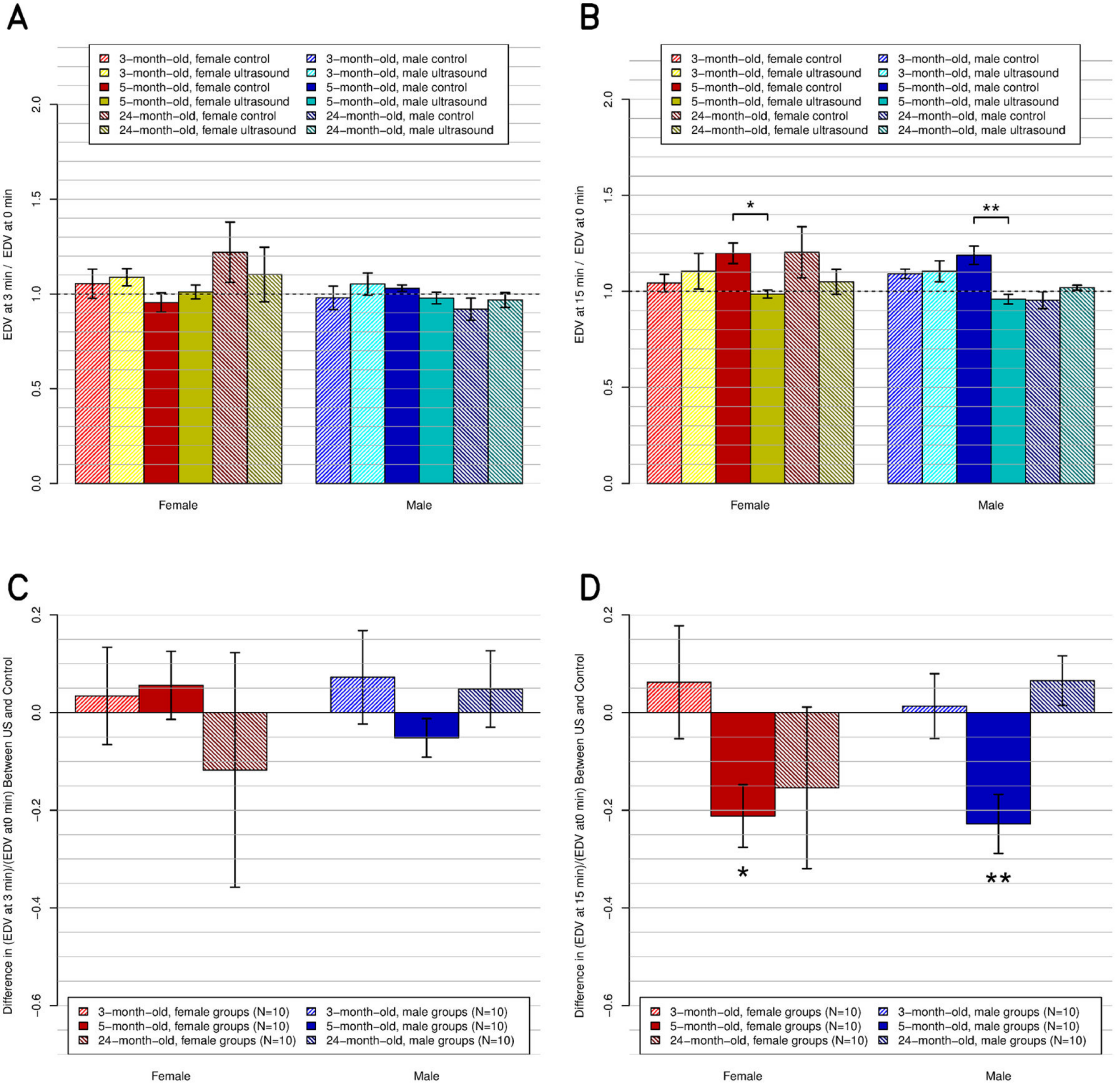
EDV, relative to preultrasound baseline, at the postultrasound times of (a) and (c) 3 and (b) and (d) 15 min. (a) and (b) All 12 subgroups individually (*N* = 5 ea.), including the six control and six experimental subgroups. (c) and (d) Same data but present the difference between the experimental subgroups and their respective control subgroups.

**Fig. 6.**
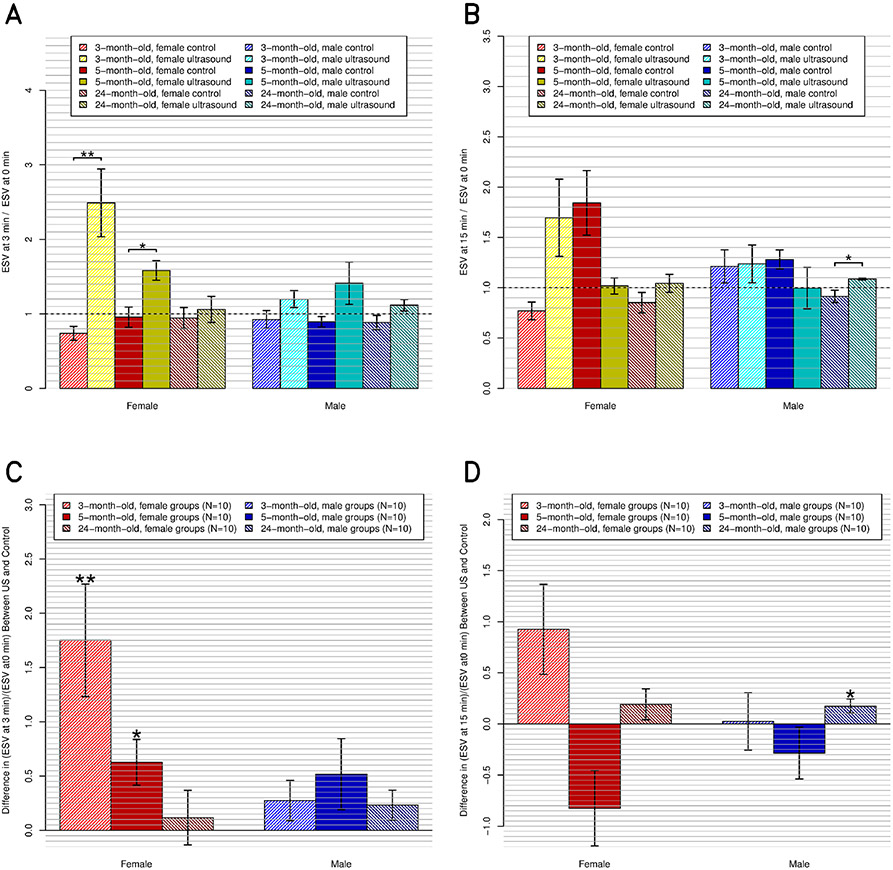
ESV, relative to preultrasound baseline, at the postultrasound times of (a) and (c) 3 and (b) and (d) 15 min. (a) and (b) All 12 subgroups individually (*N* = 5 ea.), including the six control and six experimental subgroups. (c) and (d) Same data but present the difference between the experimental subgroups and their respective control subgroups.

**Fig. 7.**
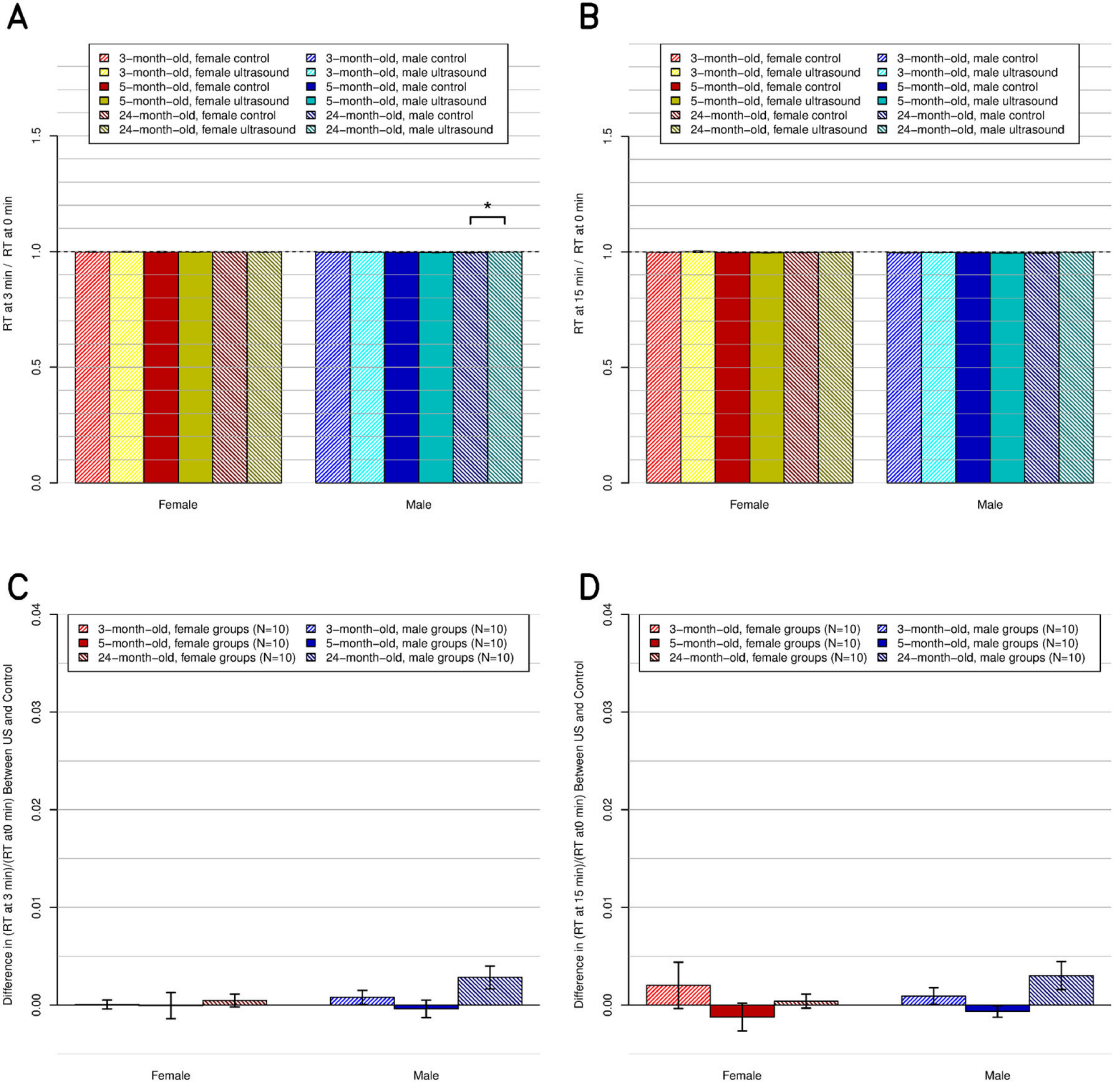
RT, relative to preultrasound baseline, at the postultrasound times of (a) and (c) 3 and (b) and (d) 15 min. Temperatures converted to kelvin before division. (a) and (b) All 12 subgroups individually (*N* = 5 ea.), including the six control and six experimental subgroups. (c) and (d) Same data but present the difference between the experimental subgroups and their respective control subgroups.

The measured physiological parameters of the rats at baseline and at 3- and 15-min post-US exposures are listed for each of the groups and are presented in Table I (three-month-old male), Table II (three-month-old female), Table III (five-month-old male), Table IV (five-month-old female), Table V (24-month-old male), and Table VI (24-month-old female). In these tables, the demographics are also listed in order of increasing weight (from 150 to 500 g). In addition, Table VII shows the significance levels for each parameter from the unpaired *t*-test between the experimental and control groups of each of the six demographics. The corresponding magnitude of change, along with its significance level, is plotted for the most relevant parameters in Fig. 3 (HR), Fig. 4 (CO), Fig. 5 (EDV), Fig. 6 (ESV), and Fig. 7 (RT). Plots for other parameters are provided in the Supplementary Material as follows: Fig. 1 (SV), Fig. 2 (EF), Fig. 3 [fractional shortening (FS)], Fig. 4 (respiratory rate), and Fig. 5 (arterial pressure).

Histological evaluation of the lung and hearts did not show any damage.

### A. Baseline

There were no statistically significant differences between sex and age for HR, CO, systolic volume, EF, EDV, ESV, respiratory rate, and arterial pressure.

### B. Heart Rate

#### 1) Within Each Demographic:

Compared to their respective control groups, US had a significant decreasing effect on HR in the three-month-old females (*p* ≤ 0.001) and the five-month-old females (*p* ≤ 0.05) at 3-min post-US exposure, but not in any other group or at 15-min post-US exposure (Fig. 3).

#### 2) Across All Groups:

At 3-min post-US exposure, the US effect (decrease of the HR) was significant (*p* ≤ 0.001), and the age and sex effects were significant (*p* ≤ 0.01). The interactions between sex, age, and US were significant (*p* ≤ 0.01). At 15-min post-US exposure, the US effect (decrease of the HR) was significant (*p* ≤ 0.01), and the sex and age effects were not significant and there were no significant interactions. The Bonferroni test showed a significant effect at 3- and 15-min post-US exposure between age (three-month-old versus six-month-old versus 12-month-old) and US (on versus off).

### C. Cardiac Output

#### 1) Within Each Demographic:

Compared to their respective control groups, US had a significant decreasing effect on CO in the three-month-old females (*p* ≤ 0.01) and the five-month-old females (*p* ≤ 0.01) and males (*p* ≤ 0.05) at 3-min post-US exposure. These effects were maintained at 15-min post-US exposure (Fig. 4).

#### 2) Across All Groups:

At 3-min post-US exposure, the US effect (decrease of the CO) and age effects were significant (*p* ≤ 0.01 and *p* ≤ 0.05, respectively) and there were no significant interactions. At 15-min post-US exposure, the US effect (decrease of the CO) was significant (*p* ≤ 0.001), and the age and sex effects were significant (*p* ≤ 0.05 and *p* ≤ 0.01, respectively). The interactions between sex, age, and US were significant (*p* ≤ 0.05). The Bonferroni test showed a significant effect at 15-min post-US exposure between sexes (male versus female).

### D. End-Diastolic Volume

#### 1) Within Each Demographic:

Compared to their respective control groups, US had no statistically significant effect on EDV in any group at 3-min post-US exposure. However, the five-month-old females (*p* ≤ 0.05) and males (*p* ≤ 0.01) had an effect (Fig. 5).

#### 2) Across All Groups:

At 3-min post-US exposure, the US and sex effects were not significant. At 15-min post-US exposure, the US effect (decrease of the EDV) and age effects were not significant, the sex effects were significant (*p* ≤ 0.05), and there were significant interactions between sex, age, and US (*p* ≤ 0.05). The Bonferroni test showed a significant effect at 15-min post-US exposures between age (three-month-old versus six-month-old versus 12-month-old) and sex (male versus female).

### E. End-Systolic Volume

#### 1) Within Each Demographic:

Compared to their respective control groups, US had a significant decreasing effect on ESV in the three-month-old (*p* ≤ 0.01) and the five-month-old (*p* ≤ 0.05) females at 3-min post-US exposure, but not in any other group (Fig. 6). At 15-min post-US exposure, those groups did not maintain their effects; however, the 24-month-old males had an effect (*p* ≤ 0.05).

#### 2) Across All Groups:

At 3-min post-US exposure, the US effect (decrease of the ESV) was significant (*p* ≤ 0.001), and the sex and age effects were not significant. There were significant interactions between sex, age, and US (*p* ≤ 0.01). At 15-min post-US exposure, there were no significant effects or interactions. The Bonferroni test showed a significant effect at 3-min post-US exposures between US (on versus off) and sex (male versus female).

### F. Rectal Temperature

Across all groups, at 3- and 15-min post-US exposure, the US (decrease of the RT) and age effects were not significant (Fig. 7). The sex effects were significant (*p* ≤ 0.05). There were significant interactions between sex, age, and US. However, the analysis within each group showed that the only demographic with a statistically significant change was the 24-month-old males and that this change was a temperature increase, not a decrease.

### G. SV (Systolic Volume, SV)

#### 1) Within Each Demographic:

Compared to their respective control groups, US did not result in a statistically significant change on SV in any group, except for the five-month-old females at 15-min post-US (*p* ≤ 0.05) (see Fig. 1, Supplementary Material).

#### 2) Across All Groups:

At 3-min post-US exposure, the US and sex effects were not significant. The age effects were significant (*p* ≤ 0.05). There were significant interactions between sex, age, and US. At 15-min post-US exposure, the US effect (decrease of the SV) was not significant, the sex and age effects were significant (*p* ≤ 0.05), and there were significant interactions between sex, age, and US (*p* ≤ 0.05). The Bonferroni test showed a significant effect at 3- and 15-min post-US exposures between age (three-month-old versus six-month-old versus 12-month-old) and sex (male versus female).

### H. Ejection Fraction

#### 1) Within Each Demographic:

Compared to their respective control groups, US had a significant decreasing effect on EF in the three-month-old (*p* ≤ 0.001) and the five-month-old (*p* ≤ 0.01) females at 3-min post-US exposure, but not in any other groups. At 15-min post-US exposure, those groups did not maintain their effects; however, the 24-month-old males had an effect (*p* ≤ 0.05) (see Fig. 2, Supplementary Material).

#### 2) Across All Groups:

At 3-min post-US exposure, in the US effect, the decrease of the EF and age effects was significant (*p* ≤ 0.001 and *p* ≤ 0.05, respectively. The interactions between sex, age, and US were significant (*p* ≤ 0.01). At 15-min post-US exposure, there were no significant effects or interactions. The Bonferroni test showed a significant effect at 3-min post-US exposures between US (on versus off).

### I. Fractional Shortening

As expected, the effects on FS paralleled the previously mentioned effects on EF.

#### 1) Within Each Demographic:

Compared to their respective control groups, US had a significant decreasing effect on FS in the three-month-old (*p* ≤ 0.001) and the five-month-old (*p* ≤ 0.05) females at 3-min post-US exposure, but not in any other group or at 15-min post-US (see, Fig. 3, Supplementary Material).

#### 2) Across All Groups:

At 3-min post-US exposure, the US effect (decrease of the FS) was significant (*p* ≤ 0.01), the sex and age effects were not significant, and there were significant between sex, age. and US interactions (*p* ≤ 0.05). At 15-min post-US exposure, there were no significant effects or interactions. The Bonferroni test showed a significant effect at 3-min post-US exposures between US (on versus off).

### J. Other Parameters

For respiratory rate and arterial pressure at both 3- and 15-min post-US exposure, there were no significant effects or interactions, overall across all groups, or within any specific demographic (see Figs. 4 and 5, Supplementary Material). Histological examination of the hearts and lungs of all animals exposed to US did not show lesions after the procedure.

## IV. Discussion

The aim of this study was to investigate the effect of US on cardiac parameters, as mediated by age and sex. This study is the first study on US-induced cardiac effects that contains data across three age groups of rats.

The present study was performed on 60 rats divided by age (3, 6, and 24 months) and sex (male and female). Considering the life cycle of female rodents, the 24-month-old females correspond to a postmenopausal female adult (about 60 years old), whereas the three- and five-month-old females correspond to a young (but post-pubescent) and young adult fertile females [26]. For comparison, in rats, sexual maturity is at seven weeks, reproductive senescence (“menopause”) is at 15–18 months, and the overall lifespan is about 2.5–3.5 years [26]. One prior hypothesis was that interaction with the sex hormones could be the major factor whether US affected cardiac parameters. This was supported by our previous results, suggesting that the effects were present only in females and that they were present in premenopausal rats, but not postmenopausal rats [25]. However, notably, in this study, we found an effect in at least one male group.

Men and women both have meaningful levels of sex hormones, including estrogen and testosterone. Literature indicates the cardioprotective role of estrogen [32], [33], and there are several proposed mechanisms, e.g., nitric oxide synthase activation or promoting the production of cardiac natriuretic hormones [34], [35]. Moreover, female sex hormones play an important role in cardiac electrophysiology [36], [37].

Our previous research demonstrated no significant changes to the intrathoracic (intercostal space) and RTs after US exposure [25]. In this present study, only RTs were monitored due to invasiveness of the intrathoracic temperature measurement; nevertheless, a similar lack of change was observed on RT in each subgroup, except for the group of 24-month-old male rats 15-min post-US exposure (sex effect, *p* ≤ 0.05, but RT increased, not decreased). These results are contradictory with our prior hypothesis that interaction with the sex hormones was the major factor determining whether US application affected cardiac parameters as the literature indicates that hormonal change is strongly associated with temperature change [38]. Studies conducted in rats and other animals showed a rise in colonic temperature at proestrus in females, and castration had the reverse impact on the temperature, the colonic temperatures of both females and males [30], [39], [40]. Furthermore, ovariectomized mice treated with estradiol demonstrated a decrease in core temperature during the light phase [9]. However, it may still be possible that there were hormonal changes and associated temperature changes in our rats, but the magnitude of the temperature change was below our statistical power to detect it in our dataset of 60 rats.

In addition, our study contained data on two different premenopausal age groups (three- and five-month-old), and the younger premenopausal age group was more affected by US than the middle-age premenopausal group (as seen specifically in HR, CO, ESV, and EF at 3-min post-US exposure). This trend suggests that there is not a strict boundary (such as menopause) before when US effects manifest fully and after when US effects are near nonexistent. Instead, it suggests that there is a more generalized age trend. This age trend was not present in the male group; the five-month-old middle-age rats were the group to show the greatest effect, particularly in arguably the most important parameter, that is, CO. In this demographic, CO was shown to be significant (*p* ≤ 0.05 at 3 min post-US exposure, *p* ≤ 0.01 at 15-min post-US exposure), while HR and SV were not. The most likely explanation for this observation is the relationship CO = HR × SV for which both HR and SV were affected but by an amount below their individual significance thresholds. However, their composite CO has the combined effects of both for which CO’s change was large enough to be statistically significant.

The exact mechanism for the change in cardiac parameters due to US exposure is not entirely understood. It has been theorized that the US effect may be weight-age dependent; our previous observations showed noteworthy differences in young female rats compared with the young males and old females rats [25]. Here, we demonstrate a similar trend and notice that the trend also matches the weight distribution: 150–200 g (three-month-old female) versus 300–350 g (three-month-old male) versus 250–300 g (24-month-old female). Moreover, differences in body weight of rats also affect the size of internal organs including the heart. The three-month-old males and the 24-month-old female rats weighted ~30% more than the three- or five-month-old rats. Because in our experiments, we used the same US transducer, it is possible that the US wavefront has an influence on more cardiac structures in the smaller rats (young and female) than the heavier ones (older and male) because of the differences in cardiac size. This would result in varying changes in the contractility of the cardiomyocytes by age and sex. In addition, older rats have more body fat in the chest area, which may result in a lower penetration depth due to increased attenuation.

Alternatively, the unfocused US transducer could be having a direct effect on cardiac tissue. Ultrasonic sonication of human cardiomyocytes *in vitro* has shown decreased intracellular calcium levels during contraction yet increased intracellular calcium in-between contractions [41]. Decreased intracellular calcium during contraction if seen *in vivo* could explain the decreased EF and CO shown in the results herein. It is also possible that the application of unfocused US stimulates proteomic changes resulting in the decrease of cardiac parameters displayed in the data. Transthoracic pulsed US to rat myocardium has been shown to transiently induce expression of inflammatory cytokines and trophic factors, such as N terminal pro b-type natriuretic peptide [42]. A transient rise in inflammatory mediators could explain why the 15-min post-US exposure group shows less significant changes in cardiac parameters in response to US stimulation.

Given the dampening effect that US has on cardiac parameters, the technology could be potentially promising for future use as an alternative or adjunct therapy for cardiac arrhythmias, and however, more studies using a larger animal model (pigs) are necessary to confirm the results. Cardiac arrhythmias are typically treated with an implantable pacemaker. Implantable pacemakers are most commonly used to treat AV nodal block, sick sinus syndrome, and atrial fibrillation [14]. Sick sinus syndrome and AV nodal block can both exhibit bradycardic features where the pacemaker is needed to stimulate the heart up to its normal rate. Negative chronotropic pacing would not be beneficial for these arrhythmias, although it could have a role in treating atrial fibrillation. A negative chronotropic effect would serve to prevent rapid ventricular response in atrial fibrillation similar to class 2 and 4 pharmaceutical antiarrhythmics [43]. Therefore, US pacing could serve as a “pill-in-the-pocket” style atrial fibrillation converter. While this is a niche role, it could still provide a benefit to those who have persistent or paroxysmal atrial fibrillation but also have contraindications for an implantable pacemaker.

In order to further access how well the effects from US pacing would translate into humans, future studies would benefit from using a more similar animal model, such as the porcine heart. The porcine heart bears a close resemblance to the human heart in terms of its coronary circulation and hemodynamic similarities [44], making it an excellent choice to continue studying the effects of US pacing on the heart. Furthermore, the study could be advanced by evaluating the different chambers of the heart individually. US that is focused on individual chambers in lieu of the entire heart may shed light on the underlying mechanism of the phenomenon we have observed. This method of only stimulating part of the heart could also eliminate the problem of varying heart sizes between females and males.

## V. Conclusion

The study has shown that pulsed US results in a significant decrease in HR, FS, and CO for young female rats, as well as a significant decrease in HR for a group of male rats. The differences between genders could be due to an interplay between various sex hormones or structural differences such as heart size and pericardial insulation. Moving forward, assessing ultrasonic pacing in a porcine heart will give insight into the viability of this technology to translate to a human model. Patients with atrial fibrillation in particular could greatly benefit from the negative chronotropic effects of ultrasonic pacing shown in the data. In addition, the feasibility of US used as a therapy for CVD, especially arrhythmias, should be further evaluated. Ultrasonic pacing has the potential to serve as a minimally invasive therapy for tachyarrhythmias, providing a nonsurgical treatment option. The goal herein was to investigate the feasibility of well-controlled unfocused pulsed US to yield cardiac pacing using specific ultrasonic exposure parameters (e.g., PRPA, PRF, and PD).

## Supplementary Material

supp3-3113867

supp1-3113867

supp4-3113867

supp5-3113867

supp2-3113867

## Figures and Tables

**TABLE I:** Measured Physiological Parameters at Baseline and at 3 and 15 min After US Exposures for Three-Month-Old Male Groups (US and Control)

	Control, 3-month-old males (N=5)			Ultrasound-applied, 3-month-old males (N=5)		
Parameter (units)	Baseline	3 Min	15 Min	Baseline	3 Min	15 Min
Heart Rate (BPM)	285.20 ± 27.29	273.60 ± 28.63	270.00 ± 28.45	246.40 ± 13.30	233.00 ± 8.62	229.80 ± 10.62
Cardiac Output (mL/min)	43.49 ± 2.96	40.62 ± 1.46	44.70 ± 2.70	41.37 ± 3.73	39.51 ± 2.85	41.55 ± 3.25
Systolic/Stroke Volume (μL)	156.08 ± 9.69	155.75 ± 15.55	172.56 ± 14.54	168.33 ± 13.76	169.98 ± 12.27	180.82 ± 11.23
Ejection Fraction (%)	87.06 ± 4.40	87.57 ± 3.24	86.68 ± 2.89	82.38 ± 2.46	80.37 ± 2.04	81.41 ± 1.60
End-Diastolic Volume (μL)	185.43 ± 20.36	182.24 ± 25.32	201.21 ± 20.45	202.33 ± 12.12	210.65 ± 11.37	221.71 ± 12.08
End-Systolic Volume (μL)	29.35 ± 12.11	26.49 ± 9.99	28.65 ± 7.93	35.80 ± 4.96	40.68 ± 3.19	40.89 ± 3.41
Respiratory Rate (/min)	41.20 ± 3.12	42.80 ± 3.03	43.00 ± 3.11	37.20 ± 2.46	35.40 ± 1.73	36.40 ± 2.05
Arterial Pressure (mmHg)	86.80 ± 2.96	87.60 ± 4.50	84.80 ± 4.53	95.20 ± 1.31	98.00 ± 2.77	95.60 ± 2.86
Rectal Tamperature (°C)	32.04 ± 0.41	31.08 ± 0.44	30.80 ± 0.48	31.84 ± 0.19	31.12 ± 0.16	30.88 ± 0.15
Fractional Shortening (%)	58.40 ± 4.66	60.03 ± 4.32	58.29 ± 3.47	53.09 ± 2.82	50.31 ± 2.13	51.41 ± 1.76

Displayed values are represented as mean ± standard error of the mean (SEM). Some of the presented is reprinted from our prior paper (Coiado and O'Brien 2017).

**TABLE II:** Measured Physiological Parameters at Baseline and at 3 and 15 min After US Exposures for Three-Month-Old Female Groups (US and Control)

	Control, 3-month-old females (N=5)			Ultrasound-applied, 3-month-old females (N=5)		
Parameter (units)	Baseline	3 Min	15 Min	Baseline	3 Min	15 Min
Heart Rate (BPM)	318.60 ± 4.46	311.60 ± 4.57	302.00 ± 4.32	306.20 ± 17.86	215.80 ± 11.00	251.60 ± 4.92
Cardiac Output (mL/min)	33.82 ± 1.54	36.41 ± 2.23	35.06 ± 2.01	30.61 ± 3.06	19.44 ± 2.48	26.08 ± 2.08
Systolic/Stroke Volume (μL)	106.12 ± 4.72	117.37 ± 8.66	115.99 ± 7.04	102.19 ± 6.24	94.07 ± 6.04	110.50 ± 7.86
Ejection Fraction (%)	85.89 ± 1.38	90.47 ± 0.89	90.00 ± 0.79	85.42 ± 2.97	70.03 ± 3.77	81.81 ± 2.71
End-Diastolic Volume (μL)	123.35 ± 3.95	129.82 ± 9.80	128.71 ± 7.14	116.50 ± 7.00	126.68 ± 9.03	126.75 ± 8.60
End-Systolic Volume (μL)	17.23 ± 1.43	12.45 ± 1.62	12.72 ± 0.82	16.75 ± 3.39	37.97 ± 5.63	23.15 ± 3.73
Respiratory Rate (/min)	44.60 ± 3.27	44.20 ± 3.33	45.00 ± 2.94	41.40 ± 2.31	38.00 ± 4.11	39.60 ± 1.31
Arterial Pressure (mmHg)	94.40 ± 7.16	92.00 ± 9.26	93.00 ± 8.73	74.20 ± 3.27	56.20 ± 8.26	64.00 ± 8.69
Rectal Temperature (°C)	32.94 ± 0.31	32.64 ± 0.23	32.42 ± 0.22	32.22 ± 0.33	31.94 ± 0.41	32.32 ± 0.90
Fractional Shortening (%)	55.83 ± 1.82	62.22 ± 1.44	61.39 ± 1.26	55.73 ± 3.32	40.17 ± 3.01	51.40 ± 2.91

Displayed values are represented as mean ± standard error of the mean (SEM). Some of the presented is reprinted from our prior paper (Coiado and O'Brien 2017).

**TABLE III:** Measured Physiological Parameters at Baseline and at 3 and 15 min After US Exposure for Five-Month-Old Male Groups (US and Control)

	Control, 5-month-old males (N=5)			Ultrasound-applied, 5-month-old males (N=5)		
Parameter (units)	Baseline	3 Min	15 Min	Baseline	3 Min	15 Min
Heart Rate (BPM)	329.00 ± 18.80	316.80 ± 15.09	319.80 ± 16.41	310.20 ± 11.90	280.80 ± 10.29	281.20 ± 6.39
Cardiac Output (mL/min)	46.30 ± 2.16	50.69 ± 2.27	60.27 ± 1.93	55.27 ± 4.12	46.55 ± 2.38	48.27 ± 2.61
Systolic/Stroke Volume (μL)	161.79 ± 4.44	169.40 ± 3.46	188.00 ± 7.64	177.30 ± 7.91	166.08 ± 7.80	171.43 ± 7.96
Ejection Fraction (%)	85.36 ± 1.85	86.92 ± 2.14	83.87 ± 2.96	86.70 ± 2.22	83.10 ± 1.80	87.43 ± 1.76
End-Diastolic Volume (μL)	190.07 ± 7.27	195.48 ± 6.43	224.20 ± 5.14	204.33 ± 6.16	200.67 ± 11.38	196.55 ± 10.09
End-Systolic Volume (μL)	28.28 ± 4.30	26.08 ± 4.91	36.20 ± 6.54	27.03 ± 4.43	34.59 ± 4.63	25.10 ± 3.96
Respiratory Rate (/min)	51.20 ± 3.65	49.62 ± 4.59	49.60 ± 2.43	53.20 ± 3.33	58.00 ± 6.40	56.00 ± 4.90
Arterial Pressure (mmHg)	90.60 ± 1.64	71.40 ± 8.33	64.00 ± 10.28	113.20 ± 2.58	98.00 ± 5.29	97.00 ± 4.87
Rectal Temperature (°C)	33.14 ± 0.16	32.16 ± 0.36	31.84 ± 0.22	32.84 ± 0.31	31.74 ± 0.28	31.34 ± 0.31
Fractional Shortening (%)	56.01 ± 2.38	58.36 ± 3.12	54.80 ± 3.34	57.99 ± 2.86	53.26 ± 2.17	58.74 ± 2.43

Displayed values are represented as mean ± standard error of the mean (SEM).

**TABLE IV:** Measured Physiological Parameters at Baseline and at 3 and 15 min After US Exposures for Five-Month-Old Femal Groups (US and Control)

	Control, 5-month-old females (N=5)			Ultrasound-applied, 5-month-old females (N=5)		
Parameter (units)	Baseline	3 Min	15 Min	Baseline	3 Min	15 Min
Heart Rate (BPM)	293.80 ± 18.34	265.60 ± 16.97	261.20 ± 16.52	277.20 ± 9.58	218.80 ± 7.14	234.60 ± 7.41
Cardiac Output (mL/min)	27.94 ± 1.50	29.08 ± 2.37	32.44 ± 2.06	35.54 ± 1.18	25.40 ± 1.97	29.64 ± 1.41
Systolic/Stroke Volume (μL)	103.74 ± 5.38	99.91 ± 7.45	112.49 ± 4.14	128.86 ± 5.73	115.58 ± 6.33	126.49 ± 5.65
Ejection Fraction (%)	83.38 ± 1.38	84.14 ± 0.96	76.27 ± 2.47	83.31 ± 0.81	74.11 ± 1.52	83.01 ± 0.92
End-Diastolic Volume (μL)	125.06 ± 8.37	118.81 ± 8.88	147.86 ± 5.25	154.52 ± 6.06	155.69 ± 6.66	152.44 ± 6.75
End-Systolic Volume (μL)	21.32 ± 3.14	18.90 ± 1.78	35.37 ± 4.26	25.66 ± 1.16	40.11 ± 2.50	25.95 ± 1.95
Respiratory Rate (/min)	36.00 ± 4.93	34.80 ± 2.86	35.60 ± 2.29	45.60 ± 2.07	43.00 ± 1.13	43.60 ± 1.82
Arterial Pressure (mmHg)	89.00 ± 9.81	86.00 ± 8.15	84.20 ± 7.57	88.40 ± 5.64	90.80 ± 5.65	90.60 ± 5.42
Rectal Temperature (°C)	31.08 ± 0.58	30.60 ± 0.26	30.38 ± 0.24	32.74 ± 0.15	32.24 ± 0.22	31.66 ± 0.19
Fractional Shortening (%)	52.76 ± 1.48	53.51 ± 1.15	45.83 ± 2.37	52.87 ± 0.95	43.67 ± 1.46	52.50 ± 1.04

Displayed values are represented as mean ± standard error of the mean (SEM).

**TABLE V:** Measured Physiological Parameters at Baseline and at 3 and 15 min After US Exposures for 24-Month-Old Male Groups (US and Control)

	Control, 24-month-old males (N=5)			Ultrasound-applied, 24-month-old males (N=5)		
Parameter (units)	Baseline	3 Min	15 Min	Baseline	3 Min	15 Min
Heart Rate (BPM)	231.40 ± 6.08	224.00 ± 6.11	218.20 ± 6.68	220.80 ± 6.15	217.00 ± 5.00	210.40 ± 4.95
Cardiac Output (mL/min)	54.54 ± 3.13	48.28 ± 4.46	49.74 ± 4.01	55.77 ± 4.54	52.10 ± 6.61	53.16 ± 4.23
Systolic/Stroke Volume (μL)	222.30 ± 6.82	206.50 ± 13.82	213.90 ± 11.05	253.10 ± 20.42	238.40 ± 27.81	251.80 ± 17.67
Ejection Fraction (%)	84.41 ± 1.50	85.26 ± 1.59	85.21 ± 1.39	78.36 ± 2.64	75.42 ± 2.93	77.03 ± 2.61
End-Diastolic Volume (μL)	263.80 ± 9.66	241.40 ± 13.56	250.80 ± 11.83	323.10 ± 23.64	315.60 ± 33.31	328.10 ± 21.65
End-Systolic Volume (μL)	41.54 ± 4.64	34.86 ± 2.79	36.89 ± 3.49	69.99 ± 10.75	77.19 ± 12.30	75.67 ± 10.89
Respiratory Rate (/min)	42.40 ± 2.38	40.80 ± 2.50	38.00 ± 2.47	43.80 ± 3.47	43.20 ± 4.19	43.80 ± 4.77
Arterial Pressure (mmHg)	67.60 ± 2.49	71.60 ± 1.95	69.80 ± 1.84	83.00 ± 7.77	80.40 ± 5.35	80.00 ± 5.40
Rectal Temperature (°C)	32.60 ± 0.62	31.36 ± 0.89	31.04 ± 0.95	31.52 ± 0.35	31.14 ± 0.28	30.88 ± 0.24
Fractional Shortening (%)	55.13 ± 1.91	56.01 ± 1.95	55.99 ± 1.75	48.99 ± 2.71	46.12 ± 2.72	47.67 ± 2.56

Displayed values are represented as mean ± standard error of the mean (SEM). Some of the presented is reprinted from our prior paper (Coiado and O'Brien 2017).

**TABLE VI:** Measured Physiological Parameters at Baseline and at 3 and 15 min After US Exposures for 24-Month-Old Female Groups (US and Control)

	Control, 24-month-old females (N=5)			Ultrasound-applied, 24-month-old females (N=5)		
Parameter (units)	Baseline	3 Min	15 Min	Baseline	3 Min	15 Min
Heart Rate (BPM)	233.80 ± 11.84	244.00 ± 9.74	249.00 ± 9.81	245.60 ± 6.33	246.80 ± 9.54	250.20 ± 9.68
Cardiac Output (mL/min)	36.21 ± 4.03	43.09 ± 4.64	43.67 ± 4.74	34.14 ± 2.61	37.13 ± 2.21	35.00 ± 0.81
Systolic/Stroke Volume (μL)	136.60 ± 16.84	164.50 ± 10.42	164.60 ± 10.30	143.40 ± 11.92	154.40 ± 9.40	149.10 ± 5.74
Ejection Fraction (%)	84.18 ± 2.65	88.77 ± 0.97	89.57 ± 0.91	83.42 ± 2.95	85.20 ± 2.06	84.55 ± 1.69
End-Diastolic Volume (μL)	160.90 ± 16.45	185.10 ± 10.83	183.90 ± 11.53	172.30 ± 13.94	181.10 ± 9.79	176.70 ± 7.92
End-Systolic Volume (μL)	24.33 ± 4.11	20.63 ± 1.84	19.26 ± 2.24	28.88 ± 6.18	26.76 ± 3.76	27.64 ± 3.77
Respiratory Rate (/min)	42.40 ± 3.23	45.60 ± 2.60	45.20 ± 2.99	37.20 ± 2.34	36.00 ± 1.90	33.80 ± 2.41
Arterial Pressure (mmHg)	83.20 ± 5.74	76.40 ± 1.51	78.20 ± 3.57	80.00 ± 3.68	76.80 ± 1.56	73.80 ± 1.51
Rectal Tamperature (°C)	32.80 ± 0.41	32.30 ± 0.52	32.06 ± 0.52	32.56 ± 0.20	32.20 ± 0.22	31.94 ± 0.22
Fractional Shortening (%)	54.62 ± 3.35	60.16 ± 1.38	61.32 ± 1.31	53.94 ± 3.44	55.70 ± 2.38	54.74 ± 2.01

Displayed values are represented as mean ± standard error of the mean (SEM). Some of the presented is reprinted from our prior paper (Coiado and O'Brien 2017).

**TABLE VII:** Summary Table of Figs. 1-5, Displaying the Significance Level of Each of the Comparisons Between Respective Experimental and Control Subgroups

		Time	3 min						15 min					
		Age	3 mo.		5 mo.		24mo.		3 mo.		5 mo.		24mo.	
Parameter	Abbr.		F	M	F	M	F	M	F	M	F	M	F	M
Heart Rate	HR		***	-	*	-	-	-	-	-	-	-	-	-
Cardiac Output	CO		**	-	**	*	-	-	*	-	**	**	-	-
Stroke/Systolic Volume	SV		-	-	-	-	-	-	-	-	*	-	-	-
Ejection Fraction	EF		***	-	**	-	-	-	-	-	-	-	-	*
End-Diastolic Volume	EDV		-	-	-	-	-	-	-	-	*	**	-	-
End-Systolic Volume	ESV		**	-	*	-	-	-	-	-	-	-	-	*
Respiratory Rate	RR		-	-	-	-	-	-	-	-	-	-	-	-
Arterial Pressure	AP		-	-	-	-	-	-	-	-	-	-	-	-
Rectal Temperature	RT		-	-	-	-	-	*	-	-	-	-	-	-
Fractional Shortening	FS		***	-	*	-	-	-	-	-	-	-	-	-

**Legend**: - Not statistically significant

* p=0.05

** p=0.01

*** p=0.001.

**Abbr.**: Abbreviation; **F**: Female; **M**: Male. Times are post-ultrasound. Significance levels are from an unpaired Student's t test between the experimental (N=5 ea.) and control (N=5 ea.) groups of the given parameter relative to pre-ultrasound application baseline value (i.e. HR15/HR0). All statistically significant changes were decreases, except those to ESV and RT, which were increases.
