# Supplementary figures and images for: Investigation of the Effects of Cardiovascular Therapeutic Ultrasound Applied in Female and Male Rats’ Hearts of Different Ages
Source: IEEE Trans Ultrason Ferroelectr Freq Control. Author manuscript; Available in PMC 2022 Feb 16. (PMC8848473; doi:10.1109/TUFFC.2021.3113867)

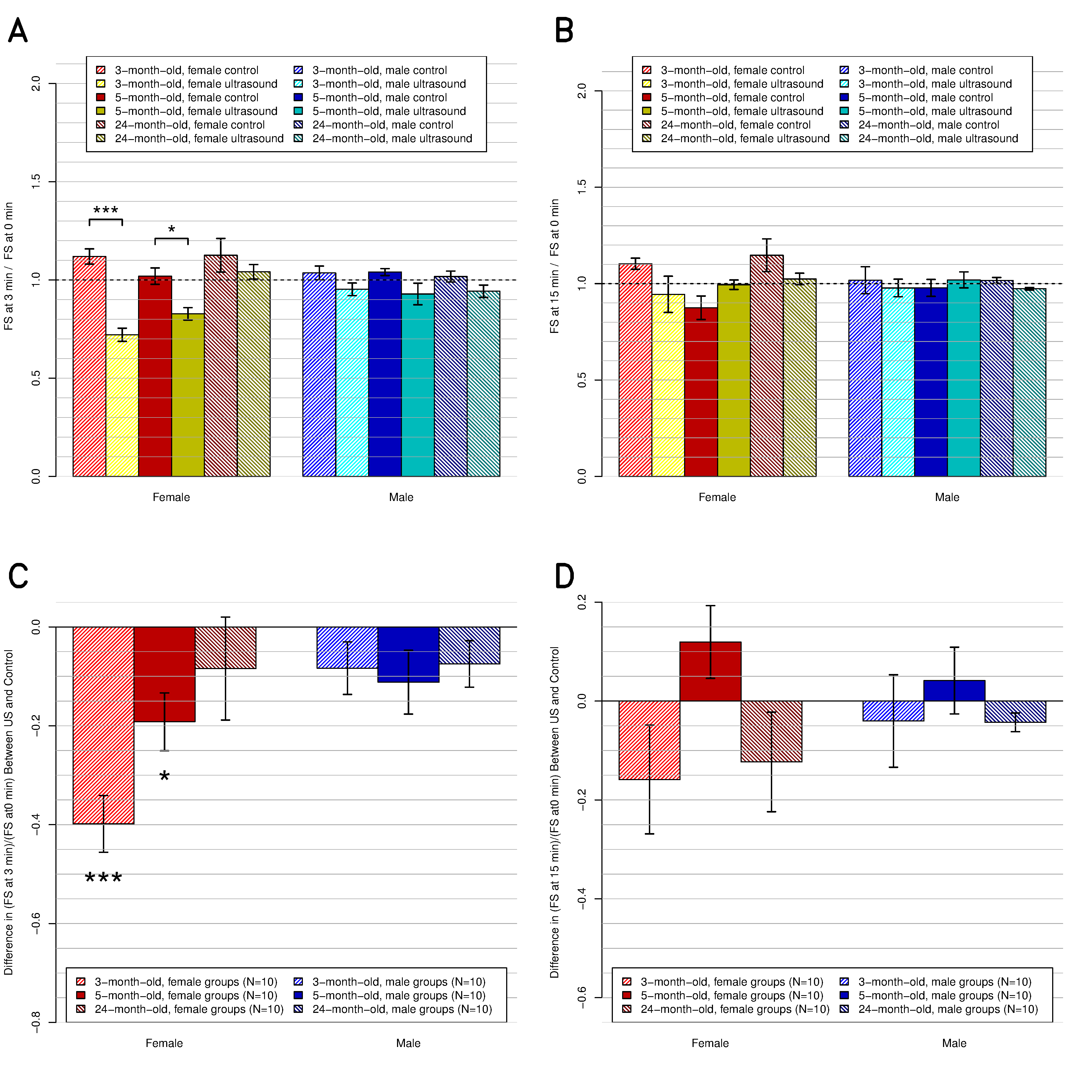

Supplement: supp3-3113867 [file NIHMS1768086-supplement-supp3-3113867.tif]

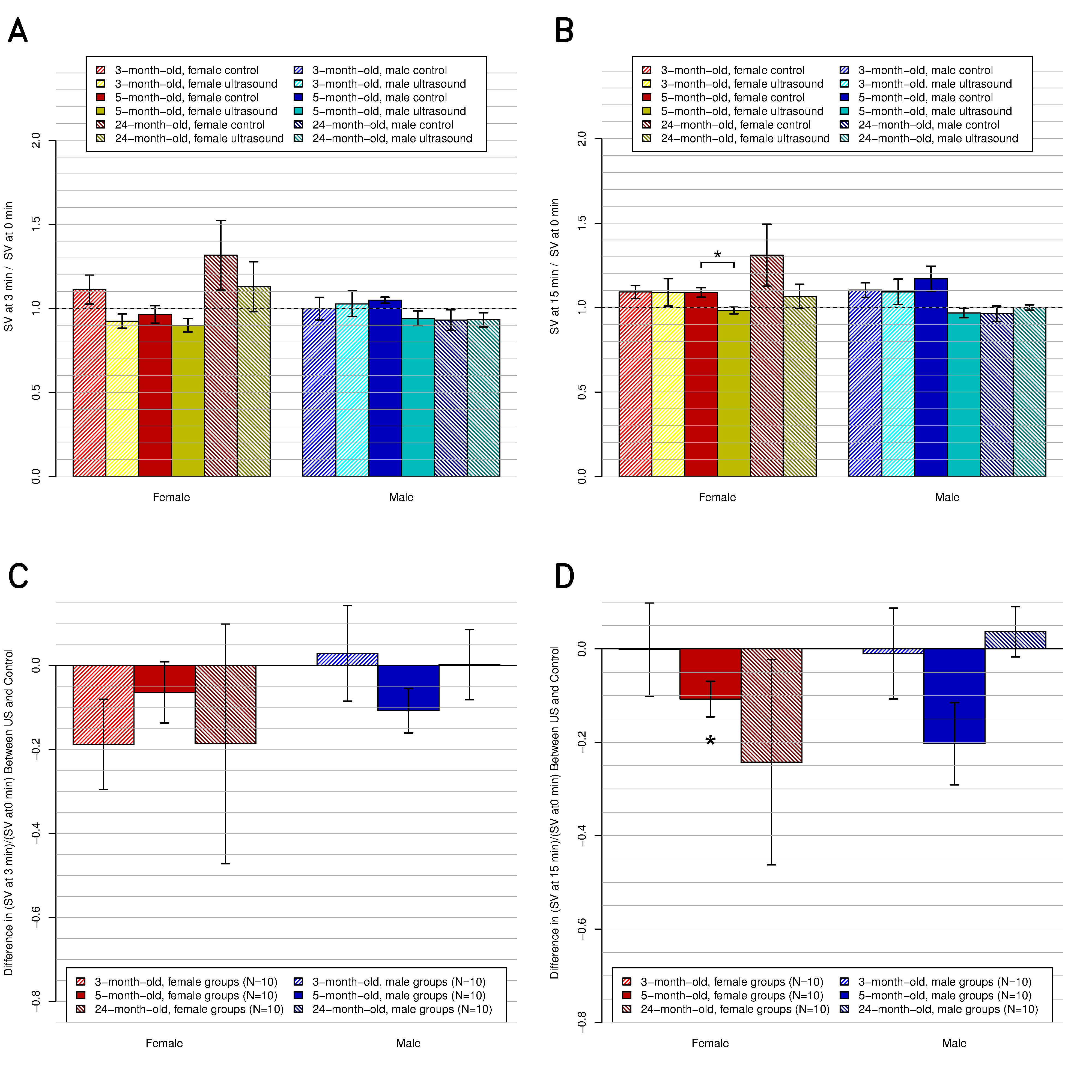

Supplement: supp1-3113867 [file NIHMS1768086-supplement-supp1-3113867.tif]

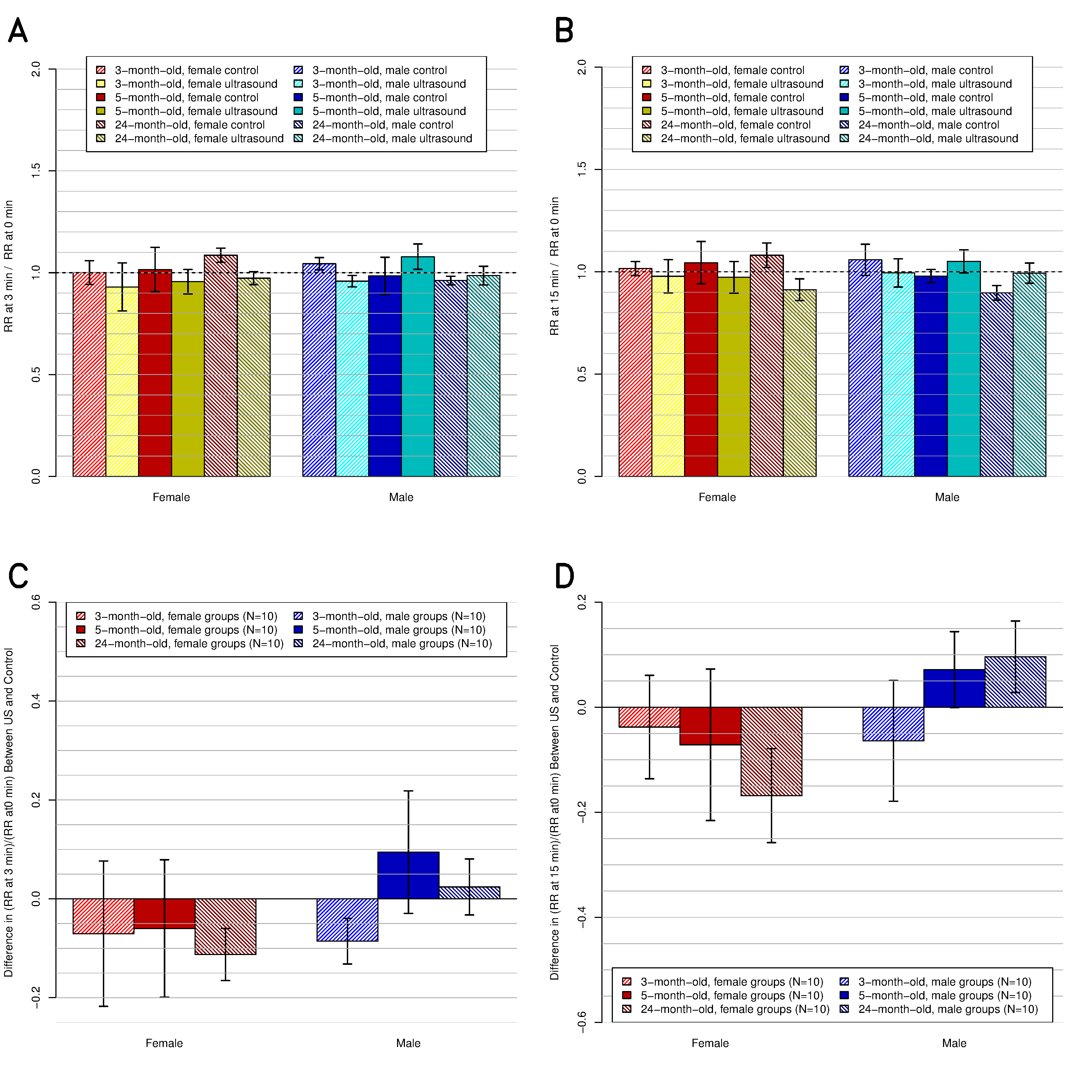

Supplement: supp4-3113867 [file NIHMS1768086-supplement-supp4-3113867.tif]

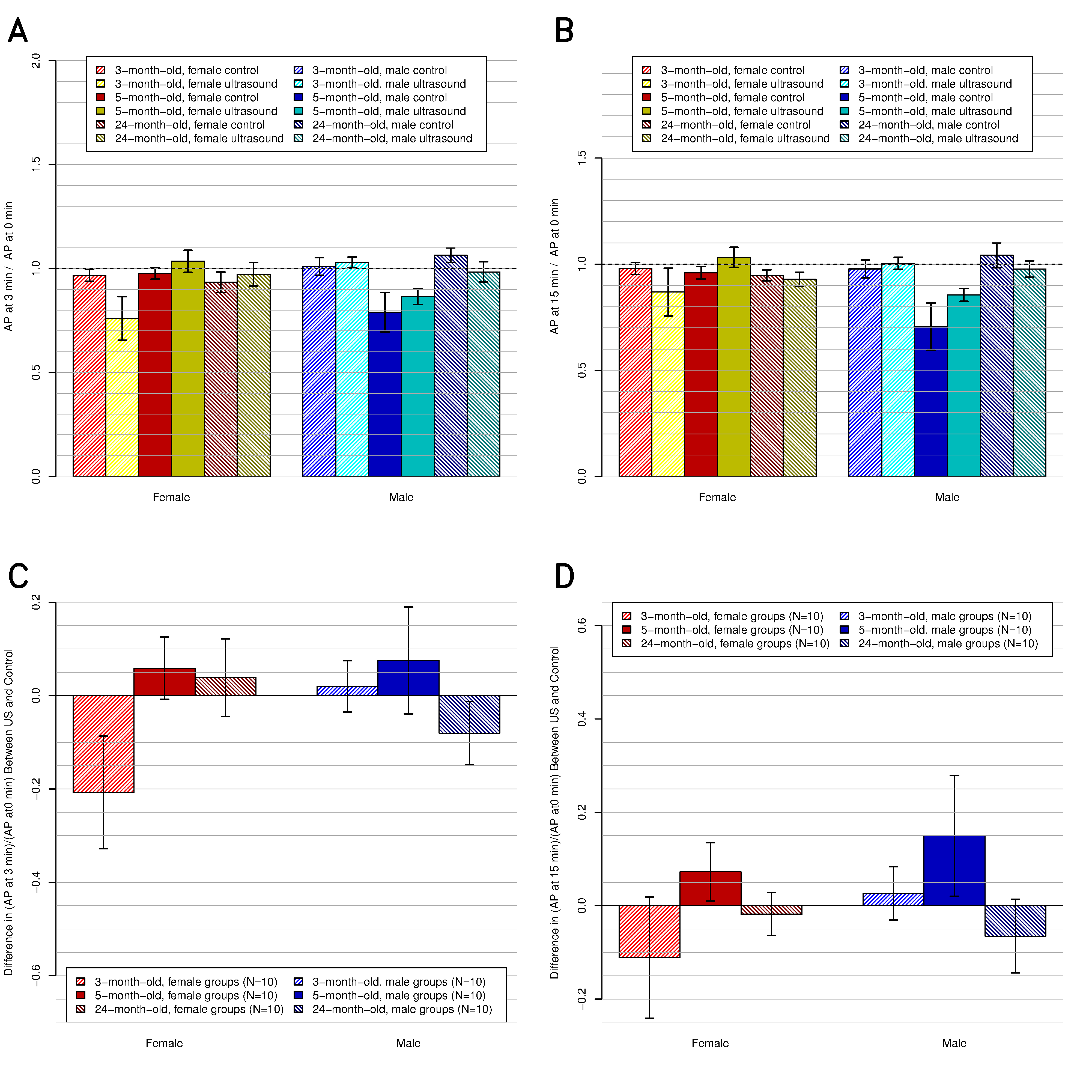

Supplement: supp5-3113867 [file NIHMS1768086-supplement-supp5-3113867.tif]

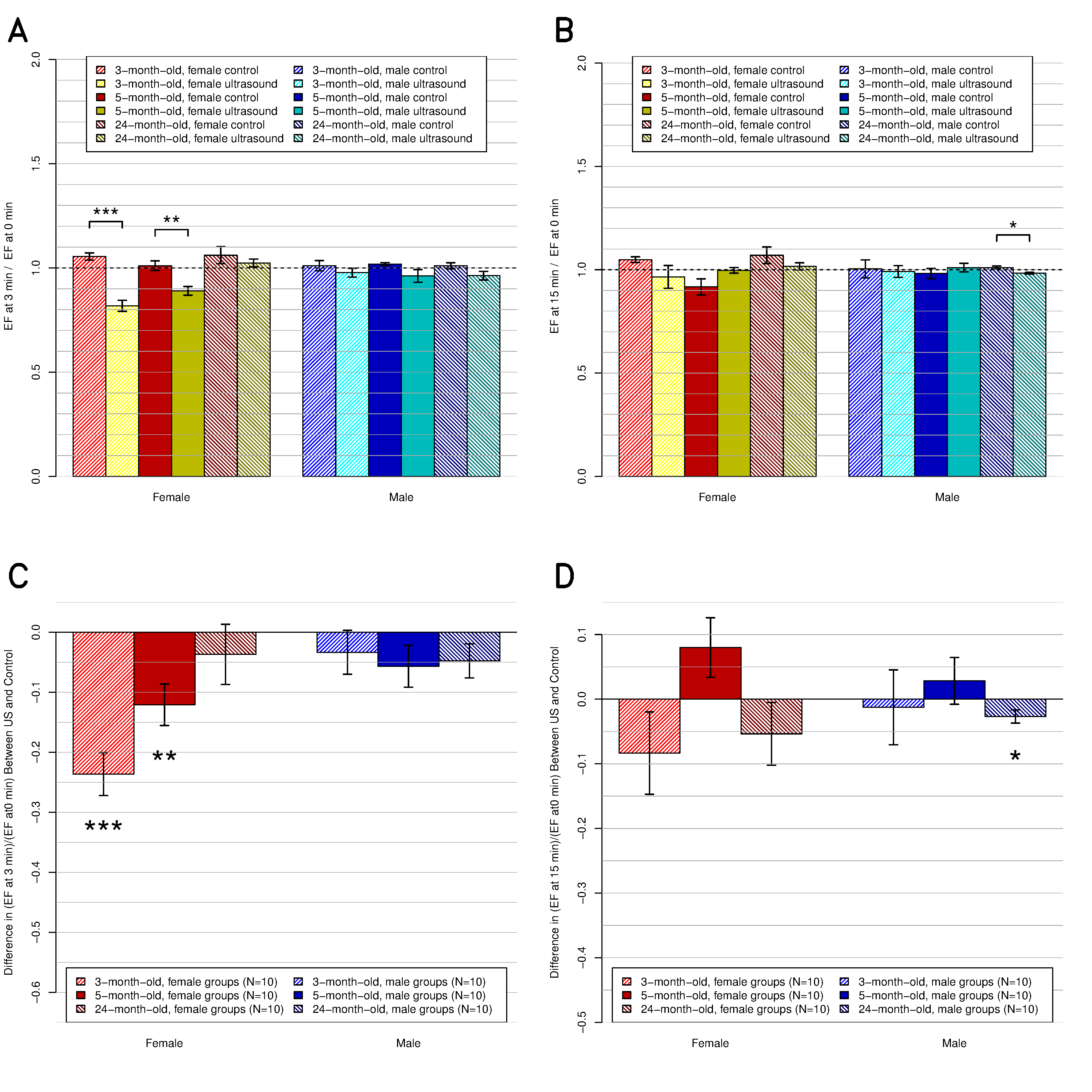

Supplement: supp2-3113867 [file NIHMS1768086-supplement-supp2-3113867.tif]
